# A Conservative Treatment Approach to Replacing a Missing Anterior Tooth

**DOI:** 10.1155/2014/625784

**Published:** 2014-08-28

**Authors:** Kunwarjeet Singh, Nidhi Gupta, Nandini Unnikrishnan, Vikram Kapoor, Dhruv Arora, Poonam K. Khinnavar

**Affiliations:** ^1^Department of Prosthodontics, Dental Materials and Implantology, Institute of Dental Studies and Technologies, Modinagar, Ghaziabad, Uttar Pradesh, India; ^2^Department of Pedodontics and Preventive Dentistry, Institute of Dental Studies and Technologies, Modinagar, Ghaziabad, Uttar Pradesh, India; ^3^Department of Prosthodontics, Dental Materials and Implantology, Bapuji Dental College and Hospital, Davangere, Karnataka, India

## Abstract

An implant-supported crown or conventionally fixed partial denture is the most common treatment modality to replace a missing anterior tooth but a more conservative approach, with a fiber reinforced composite resin FPD, can be used to replace a missing anterior tooth in young patients or when the patient does not agree for an implant, or conventional FPD or RPD therapy. It is an esthetic, conservative single sitting chairside procedure which can be used as a definitive treatment alternative in certain clinical situations for esthetic and functional replacement of a missing anterior tooth. To achieve desirable results, putty matrix was used for proper positioning of the pontic during direct fabrication of FRCFPD.

## 1. Introduction

The esthetic and functional rehabilitation of a missing anterior tooth is one of the greatest challenges that the dentist faces. The procedure becomes difficult when the missing tooth cannot be replaced by an implant-supported prosthesis or a conventional fixed dental prosthesis due to a local bony defect, inadequate volume of bone in the edentulous area, occlusal function, systemic disorders, or the socioeconomic status or unwillingness of the patient to experience invasive implant surgery or the preparation of natural teeth for retainers. The fiber reinforced composite resin FPD (FRCFPD) can be considered a viable alternative in such situations or in those in which conservative preparation is needed [1].

Fiber reinforced composite (FRC) materials consist of glass, carbon, or polyethylene fibers contained within a resin matrix. The type of fiber, the fiber architecture, and the quality of the fiber/matrix coupling determine the mechanical properties of the material. Laboratory studies have shown that FRC materials exhibit flexure strength that is comparable to or greater than that of metal alloys [2] but they exhibit a lower flexure modulus [3]. Clinical usage and clinical research have shown that FRC prostheses can be used to satisfactorily restore or replace teeth with fixed prostheses [4, 5].

The polyethylene fiber reinforced fixed dental prostheses consist of a fiber reinforced composite (FRC) substructure veneered with a composite material and a ceramic, acrylic, or composite resin pontic. The successful esthetic and functional rehabilitation of missing tooth with direct FRCFPD depends on accurate positioning of pontic in patient's mouth. It is difficult to hold the pontic in proper position with instrument or fingers during direct fabrication of FRCFPD. For accurate positioning, stabilization of pontic is very important which can be achieved with putty matrix. Putty matrix maintains pontic in accurate mesiodistal, labiolingual, and cervicoincisal position during direct fabrication of FRCFPD.

This clinical procedure with putty matrix can be used as a long term alternative for successful rehabilitation of a missing tooth in patients with vital intact abutment teeth, short edentulous span (1 or 2 missing teeth), and minimum dynamic occlusal contacts on the abutment teeth [6, 7]. The advantages of such a prosthesis are minimal preparation of lingual and proximal surfaces of abutment teeth and supragingival finish lines with good tissue tolerance and reduced cost and chairside time. Long term durability and success of the FRCFPD depend on careful patient selection, proper design, precise preparation, appropriate selection of materials, and bonding techniques. Sufficient horizontal overlap and minimum vertical overlap must be present between maxillary and mandibular anterior teeth to minimize functional stresses and occlusal loading of the pontic, to reduce the chances of debonding. The supporting abutment must be periodontally sound with adequate bone support and no mobility. If primary abutments are weak, secondary abutments must be used to ensure long term success of the prosthesis.

Polyethylene fibers with a custom made composite resin, PFM, or all-ceramic pontic can be successfully used for fabrications of FPD. High strength polyethylene fibers can be used to increase the strength of provisional acrylic or composite resin crowns and FDP, orthodontic retainers, periodontal splints, dentures, and occlusal guards and for repair and reinforcement procedures [8].

## 2. Case Report

**Figure 1:**
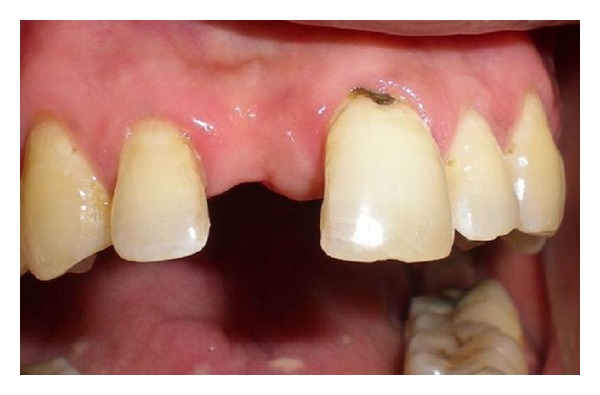
Missing maxillary right central incisor prior to treatment.

An 18-year-old male patient reported to the Department of Prosthodontics and Dental Materials at Institute of Dental Studies and Technologies, with a chief complaint of unsatisfactory esthetics due to a missing maxillary right central incisor. The intraoral examination revealed a healthy dentition with minimal calculus and stains on the lingual and palatal surfaces of the teeth with healthy periodontal tissues and cervical dental caries on the labial aspect of the left maxillary central incisor (Figure 1), which was later restored with light cure composite resin (Ceram x Duo, Dentsply, Germany). The patient had stable maximum intercuspation and canine guided occlusion with approximately 2.5 mm vertical and horizontal overlaps (Figure 5). There was no evidence of bruxism or wear facets on the occlusal surfaces. Radiographic evaluation revealed a sound abutment and adequate crown-root ratio with no residual ridge deficiency. On the basis of clinical and radiographic findings, the patient was presented with several treatment options which included an implant-supported crown, conventional fixed partial denture, resin-bonded fixed partial denture, and a polyethylene fiber reinforced FPD with ceramic or composite resin pontic. The implant-supported crown was rejected because of the time duration and the necessity of surgical intervention. Similarly a conventional FPD was rejected as the patient did not want to sacrifice his natural teeth. Resin bonded FPD was less invasive as compared to conventional FPD; however, the patient was concerned about the esthetic aspect of the metal framework and involvement of almost the entire palatal surface of abutments for placement of retainers. The patient thus opted for a FRCFPD with composite resin pontic as this would require only a single sitting procedure with minimal tooth preparation on the palatal surfaces of abutment teeth, ruling out surgical and complete abutment preparation procedures. During four-year followup, no debonding was observed and patient was satisfied with the outcome.

## 3. Clinical Procedure

**Figure 2:**
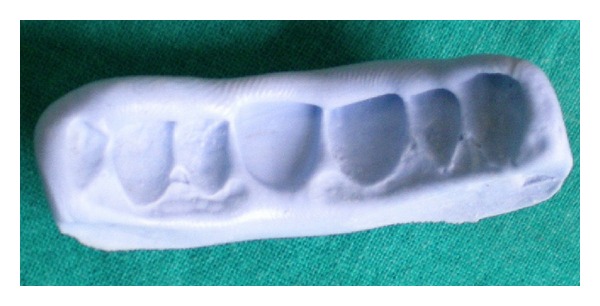
Putty index fabricated on cast after stabilization of pontic on cast with wax.

After oral prophylaxis and restoration of cervical caries of the maxillary left central incisor with light cure composite resin (Ceram x Duo, Dentsply, Germany), a preliminary maxillary impression was made with polyvinyl siloxane elastomer of putty consistency (Aquasil soft putty/regular set, Dentsply, Germany). The composite resin pontic was fabricated using an incremental buildup technique on the impression of the maxillary left central incisor (Ceram x Duo, Dentsply, Germany) followed by light curing. Ceram x Duo is a nanoceramic restorative material supplied in dentin and enamel shades which facilitates shade matching. The pontic obtained by this technique replicates the maxillary left central incisor which was made to resemble the maxillary right incisor with minor modifications using composite resin. A properly finished and polished modified ridge lap pontic design was made. After fabrication of the pontic, the same impression was poured with die stone to obtain a cast. The pontic was then placed on the cast in accurate buccolingual, mesiodistal, and cervicoincisal position as per the esthetics and stabilized with wax from the palatal side. A putty matrix/index (Figure 2) was then made with polyvinyl siloxane elastomeric putty on the cast with the pontic in position. This matrix was used for accurate positioning of the pontic in the patient's mouth (Figure 4) and it helps to minimize the pressure exerted by the pontic on the edentulous area.

**Figure 3:**
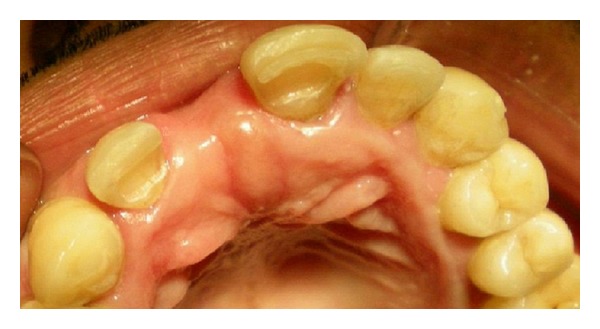
Slots prepared at palatal surface of abutments.

Palatal grooves, approximately 3 mm wide and 1.5 mm deep, involving at least three-quarters of the mesiodistal width of the abutments, were prepared with round and inverted cone diamond rotary burs, in the patient's mouth. A similar size groove involving the whole of mesiodistal width of the pontic was prepared on the pontic (Figure 3). The grooves prepared on abutments and pontic should be at the same level.

The distance between the grooves was measured and a piece of the fiber ribbon (Perfect Splint, Hager Werken, Germany) was cut with the same dimensions as the space between two grooves. The bonding agent (Prime and Bond NT, Dentsply, Germany) was applied on the cut piece of fiber ribbon and kept aside. The fiber impregnated with bonding agent should be kept out of the dental light until used. One should avoid touching the fiber ribbon after it is wetted with bonding agent via the fingers, because any contact can contaminate its reactive surface layer.

**Figure 4:**
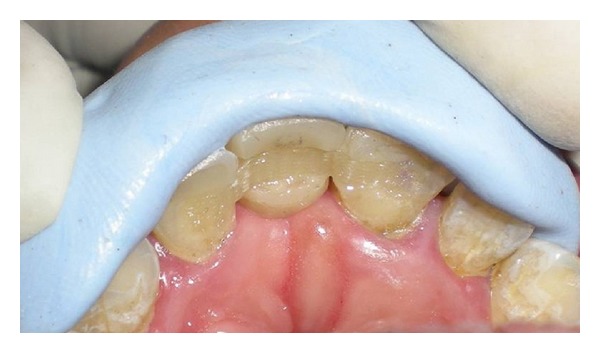
Fiber reinforced composite resin placed in slots after stabilization of pontic with putty index.

**Figure 5:**
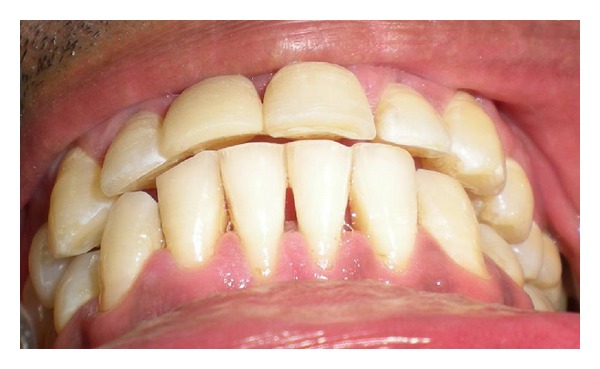
Incisal view of definitive prosthesis.

**Figure 6:**
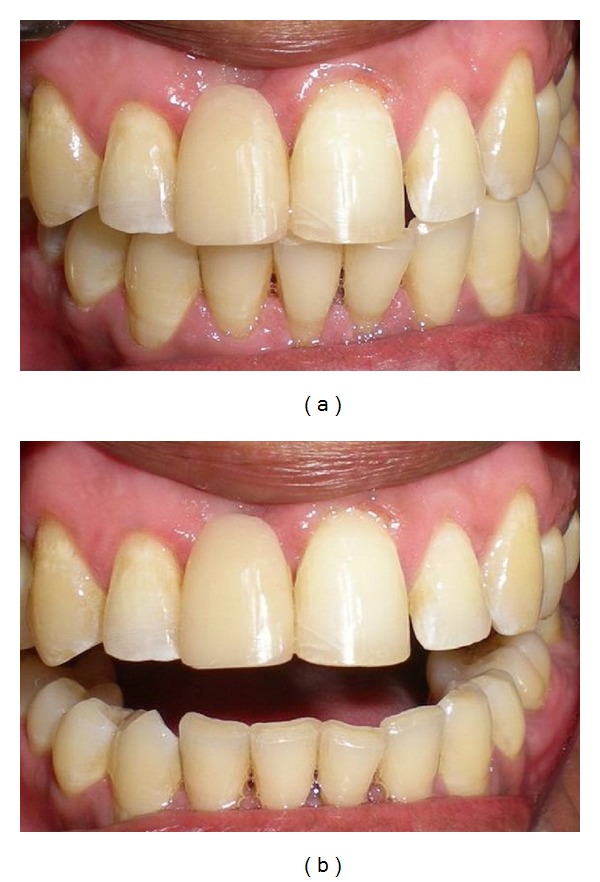
(a) and (b) Facial view of definitive prosthesis.

The prepared slots and mesioproximal surfaces of both the abutments were then etched with 37% phosphoric acid (Scotchbond multipurpose etchant, 3 M ESPE, USA) for 15 seconds. After thoroughly rinsing and drying, a bonding agent (Prime and Bond NT, Dentsply, Germany) was applied with a microbrush applicator on both the prepared abutment areas and on the groove on the pontic. Excess bonding agent was removed with the brush tip and by gently blowing with air. The bonding agent was light cured for 15 seconds. The pontic was then placed in the patient's mouth and held in accurate position with the putty index (Figure 4). Ceram x Duo composite of the selected shade was first placed properly in the grooves on the abutments and on the pontic followed by placement of the cut piece of fiber ribbon along the grooves using composite placement instruments. The excess composite which flows into the proximal embrasures should be removed carefully before starting polymerization. The composite with fiber ribbon was carefully polymerized with a Quartz tungsten halogen light curing unit for 40 seconds each from the buccal and palatal surfaces of both abutments and from the lingual surfaces of pontic. Finally a flowable composite (tetric flow, Ivoclar Vivadent) was placed over polyethylene fibers, giving a smooth, glossy appearance on palatal surfaces. Occlusion was evaluated and premature contact was eliminated (Figure 6(a)). Figures 5, 6(a), and 6(b) show incisal and labial views of definitive prosthesis, respectively. The patient was very satisfied with the final outcome and was advised to maintain oral hygiene with proper brushing technique. Three-year successful clinical followup of the patient was done. During followup, no debonding was observed and only slight wearing of the composite resin was observed on the palatal side of the abutments which was directly repaired in the patient's mouth.

## 4. Discussion

Replacement of missing teeth with a FRCFPD with a composite resin pontic is a simple, single visit chairside procedure. This report describes the replacement of the right maxillary central incisor with an esthetic and conservatively fixed partial denture and a technique for fabrication of a composite resin pontic and putty index for accurate positioning of the pontic in the patient's mouth.

Fiber reinforced composite resin FPD and resin bonded FPD can be considered conservative approaches for replacing missing anterior tooth in certain favorable clinical conditions. A common problem with metal ceramic resin bonded FPD has been the grayish discoloration of the incisal third of the abutment teeth due to cast metal lingual retainers [9] and debonding of metal retainers from tooth if careful execution of bonding technique is not done. The fiber reinforced composite resin FPD require only preparation of palatal slots on the middle of the palatal/lingual surface. The retention of these FPD depends on the proper placement of fiber framework in the grooves and careful bonding procedure. The incidences of debonding are less when fiber framework is properly placed in the grooves and bonding procedure is carefully executed. In addition to above-mentioned advantages, other advantages include completion of procedure in single appointment, low cost, and less invasiveness, and repairs can be carried out directly without the need for any complicated techniques or materials. Adjustments to the design, esthetic details, and occlusal relationships may be carried out immediately or with a minimum of time during followup appointments.

All-ceramic, porcelain fused to metal (PFM), acrylic resin or a composite resin pontic can be used with fiber reinforced composite resin. An all-ceramic and PFM pontic was not selected by the patient because of the cost factor. The bonding of these pontics with fiber reinforced composite requires etching with 10% hydrofluoric acid and a silane coupling agent which increases the cost. The acrylic resin denture tooth is not recommended as a pontic with fiber reinforced composite resin because of the unpredictability of bonding acrylic with composite resin. It is mainly used for fabrication of provisional crowns and FPD. The advantage of a composite resin pontic is that it can be fabricated easily by incremental build-up of composite in the impression of an adjacent central incisor made in putty of elastomer impression material in a single visit. After removal from the impression, the pontic can be made to match in shade and shape the adjacent central incisor by modification with composite resin of the selected shade and finishing and polishing. A putty index should always be used for accurate positioning of the pontic in the patient's mouth. This also helps in maintaining passive contact of the pontic with underlying tissues.

With advancements in composite resin, the new generation composite resins have very good wear and stain resistance. The new generation nanoceramic composite resins are available in different enamel and dentin shades, so shade matching and characterization of the pontic are easy and esthetic results obtained are really good. These prostheses can be successfully used as short term alternatives for replacement of missing anterior teeth in young patients when conventional FPD are contraindicated. The conservative preparation, advancement in bonding systems, and reported success suggest that this prosthesis can be used as a long term definitive alternative in situations similar to the case described. Long term success depends on proper abutment selection, slot preparation, careful bonding technique, and type of occlusion. There should be no contact on the pontic, sufficient horizontal overlap, and minimum vertical overlap.

This technique is simple, easy, and less time consuming than other approaches. It is an affordable and quick solution for the patients who reject more invasive treatments. Further studies are needed to evaluate the long term usefulness of the polyethylene fiber reinforced composite resin fixed partial denture with a composite resin or all-ceramic pontic.

## 5. Conclusion

This report describes a clinical procedure for fabrication of a composite resin pontic and polyethylene fiber reinforced fixed partial denture. It also describes a technique for fabrication of a putty index made with polyvinyl siloxane elastomeric impression material which is important for accurate positioning of the pontic in the patient's mouth. This technique can be successfully used as a short or long term alternative for replacement of missing anterior tooth in a young or adult patient.
